# Six-Week Problem Area–Concordant vs 8-Week Problem Area–Discordant Group Interpersonal Psychotherapy: A Randomized Clinical Trial

**DOI:** 10.1001/jamanetworkopen.2025.5242

**Published:** 2025-04-16

**Authors:** Rosco Kasujja, Peter Birungi, Kasturi Bhamidipati, Frey Assefa, Hae-Young Kim, Katia M. Peterson, Brandon A. Kohrt, Anna Bershteyn

**Affiliations:** 1 Innovation Lab, StrongMinds International, Kampala, Uganda; 2 Department of Mental Health, Makerere University, Kampala, Uganda; 3 Department of Population Health, New York University Grossman School of Medicine, New York, New York; 4 In Situ Research LLC, Washington, DC; 5 Center for Global Mental Health Equity, Department of Psychiatry and Behavioral Health, George Washington University, Washington, DC

## Abstract

**Importance:**

Depression is a prevalent mental health condition contributing to morbidity worldwide. The World Health Organization (WHO) recommends group-based interpersonal psychotherapy (IPT-G) for first-line depression treatment in resource-constrained settings. Standard of care in the study context is 8 to 12 weekly sessions in groups with a mix of depression problem areas (eg, grief, life changes, loneliness, conflict).

**Objective:**

To investigate whether grouping participants with a common depression problem area (problem area–concordant) using shortened IPT-G (6 sessions) is noninferior to grouping participants with a mix of problem areas (problem area–discordant) using standard IPT-G (8 sessions) in Uganda.

**Design, Setting, and Participants:**

This noninferiority randomized clinical trial included adults 18 years or older in central Uganda with 9-item Patient Health Questionnaire (PHQ-9) scores of 10 or greater, indicating symptoms consistent with probable depression. Assessors were masked to treatment arm. Data were accrued from October 31, 2022, to March 24, 2023.

**Interventions:**

Participants were randomized 1:1 to 6-session problem area–concordant or 8-session problem area–discordant IPT-G.

**Main Outcome and Measures:**

The primary outcome was PHQ-9 score reduction at 3 months. Secondary outcomes were treatment response (PHQ-9 5-point, 10-point, and 50% score reduction), reduction in disability (WHO Disability Assessment Schedule 2.0), and improvement in subjective quality of life (WHO Quality of Life tool).

**Results:**

Among 328 enrolled participants (303 [92.4%] female; mean [SD] age, 42.3 [15.2] years), retention was high, with 321 [97.9%] undergoing assessment at the end of therapy and 292 [89.0%] at 3-month follow-up. From baseline to the end of therapy, PHQ-9 scores dropped a mean (SD) of 15.2 (5.1) points in the problem area–concordant arm and 13.3 (5.3) points in the problem area–discordant arm. Problem area–concordant 6-week IPT-G was noninferior (*P* < .001) at end of therapy and 3 months post therapy. Compared with the 8-week problem area–discordant arm, posttherapy PHQ-9 scores in the 6-week problem area–concordant arm were 1.86 (95% CI, 0.74-3.00) points lower (*P* = .001). At 3 months, PHQ-9 scores were 1.98 (95% CI, 0.60-3.36) points lower (*P* = .005). Disability score reduction was significantly larger post therapy in the 6-week arm compared with the 8-week arm (2.70 [95% CI, 0.95-4.44] points) but not significantly different between arms after 3 months. Quality of life scores across all domains were not significantly different between arms at end of therapy and 3 months post therapy.

**Conclusions and Relevance:**

In this randomized clinical trial, 6-week problem area–concordant IPT-G was noninferior to 8-week problem area–discordant IPT-G for reducing depression symptoms, with similar to larger improvements in disability and quality of life. Problem area–concordant group therapy appears to be a promising approach to increase efficiency and scalability of depression treatment.

**Trial Registration:**

Pan African Clinical Trials Registry Identifier: PACTR202306771120632

## Key Points

**Question:**

Does shortened (6-session) interpersonal group psychotherapy (IPT-G) with participants experiencing a common depression problem area (problem area–concordant) provide noninferior reduction in depressive symptoms, compared with 8-session IPT-G with participants experiencing a mix of problem areas (problem area–discordant)?

**Findings:**

In this randomized clinical trial of 328 participants, problem area–concordant 6-week IPT-G was noninferior to 8-week problem area–discordant IPT-G. Nine-Item Patient Health Questionnaire scores dropped by nearly 2 additional points compared with the 8-week group, and this difference was maintained after 3 months.

**Meaning:**

Shortening IPT-G while grouping clients with a common depression problem area offers a promising approach to increase depression treatment efficiency and scalability.

## Teaser

This noninferiority randomized clinical trial, conducted in Uganda, compares 8-week problem area–discordant group-based interpersonal psychotherapy for depression with a 6-week version that groups clients with the same depression problem areas.

## Introduction

Depression significantly contributes to morbidity in adolescents and adults,^1^ ranking among the top 10 contributors to the global disease burden^2^ due to its high prevalence and substantial impact on quality of life.^3^ Approximately 5% of adults experience depression globally,^4^ with higher rates in populations such as refugees,^5^ people living in poverty,^6^ and people living with HIV.^7^ In African low- and middle-income countries, where rates of extreme poverty^8^ and HIV^9^ are among the highest globally, approximately 12% to 17% of all adults report experiencing depressive symptoms in the past 2 weeks.^10^ In Uganda, depression prevalence is approximately one-third of adults and even higher in some communities (eg, two-thirds of refugees).^11^

Structured psychological interventions, such as cognitive behavior therapy and interpersonal therapy (IPT), have demonstrated efficacy for reducing depressive symptoms.^12^ In resource-constrained settings, the World Health Organization (WHO) recommends interpersonal group psychotherapy (IPT-G) as an effective first-line treatment for moderate to severe depression.^13^ IPT-G, adapted from the individual IPT model, is a structured, therapist-facilitated series of group therapy sessions focused on recognizing the problem areas and symptoms of depression, setting personalized goals, and assessing progress toward symptom reduction.^14^ It addresses depression by targeting interpersonal problem areas in the context of shared experiences.

IPT-G is well suited for settings with high depression prevalence because it can be delivered directly in communities and scaled with limited resources. After individual screening for depression symptoms, clients are grouped for therapy sessions, improving scalability—given the large numbers of clients and paucity of mental health therapists—while also enhancing social support during therapy.^15^ Therapy has also been shortened from 16 to 8 weekly sessions to reduce the burden on clients and therapists.^16^ These adaptations have been shown to maintain effectiveness while supporting scalability and cost-effectiveness.^17,18^

Despite these innovations in scaling IPT-G, the number of people in need of depression therapy far outstrips delivery capacity, necessitating further adaptation to accelerate scale-up in resource-limited settings. Current standard of care in the study context is 8 to 12 weekly sessions in groups with a mix of depression problem areas: grief or loss of a loved one, life changes, loneliness or isolation, or interpersonal conflict or disagreement. Eight weekly sessions, though half of the 16 sessions used in earlier IPT-G approaches,^14,19^ limits the number of clients therapists can treat.^15^ Additionally, clients experiencing depression risk factors, such as poverty and refugee status, who already have a high cognitive load in day-to-day life,^20,21^ struggle to attend the large numbers of sessions due to the added burden.^22^ Adaptations are needed not only to reduce IPT-G costs and increase scalability but also to make therapy less burdensome, both logistically and cognitively.

To enhance the efficiency and scalability of IPT-G in resource-constrained settings, we shortened the 8-week problem area–discordant IPT-G protocol into a 6-week problem area–concordant version by grouping clients with the same depression problem areas and reducing the number of sessions by 25%. Given that mixed groups may distract from an individual’s issues, group homogeneity is postulated to enhance perceived relevance, strengthening peer empathy and social support. We hypothesized that this adapted IPT-G would be noninferior to 8-week problem area–discordant IPT-G.

## Methods

Participants received a participant study information sheet or a verbal study description from a research assistant and provided written informed consent. The study was approved by the Research Ethics Committee of the Makerere University School of Health Sciences, Kampala, Uganda, and was conducted under the oversight of an independent data safety and monitoring committee. Reporting of results for this study followed the 2010 Consolidated Standards of Reporting Trials (CONSORT) updated guidelines for reporting parallel group randomized trials and the CONSORT Extension for Reporting of Noninferiority and Equivalence Randomized Trials.^23^ The study protocol is found in Supplement 1.

### Study Design and Participants

We conducted an individually randomized clinical noninferiority trial designed to have 80% power to detect a noninferiority margin of 1 point on the 9-item Patient Health Questionnaire (PHQ-9)^24^ (eMethods in Supplement 2). Eligible participants were adults 18 years or older, living in the Buikwe and Kayunga districts of central Uganda, and screened for depression in their communities. Further details on the study setting and recruitment are provided in the eMethods in Supplement 2.

Eligibility criteria included a baseline PHQ-9 score of at least 10, indicating symptoms consistent with probable depression,^25^ and the ability to speak Luganda or English and to provide informed consent. Participants were excluded if they had a previous diagnosis of intellectual disability, were receiving treatment for a psychiatric disorder, reported frequent suicidal ideation (more than half the days or nearly every day in the past 2 weeks), or reported having visual, speech, and hearing impairments with no aids. Individuals deemed to require higher levels of psychiatric care than community-based IPT-G were referred to local services for assessment and intervention.

### Randomization

Eligible participants were stratified by the 2 districts and then individually randomized in a 1:1 ratio to either problem area–concordant 6-week or problem area–discordant 8-week IPT-G. Both therapists and participants were informed of randomization at the time of group assignment, as this was required to schedule the number of sessions. Independent outcome assessors were masked to the study arm.

### Grouping

After randomization, participants in the 6-week arm were assigned to therapy groups based on their problem areas, while those in the 8-week arm were assigned to therapy groups irrespective of their problem areas. Depression problem areas were assigned based on participant interview in categories of (1) grief or loss of a loved one, (2) life changes, (3) loneliness or isolation, or (4) interpersonal conflict or disagreement, following standard IPT-G pregroup interview procedures.^13^ Multiple problem areas could be recorded for the same participant.

Participants in the 6-week problem area–concordant arm were grouped by proximity to their homes and shared problem areas. Grouping in this arm was performed by placing participants with only 1 depression problem area into groups by problem area. Then, participants with multiple problem areas were placed into one of these groups that focused on at least 1 of their problem areas.

Participants who could not be placed in a therapy group matching their reported problem area(s) due to the lack of a group within travel distance of their home were placed in groups that focused on the problem area of life change, the broadest problem area covered by IPT-G. Participants in the 8-week problem area–discordant arm were grouped by location and order of enrollment, irrespective of their problem area, as per standard of care.

### Intervention

The study used the group interpersonal psychotherapy (IPT) for depression protocol^13^ for 8-week IPT-G and shortened it to a 6-week, single–problem area IPT-G protocol. Unlike 8-week IPT-G, in which all 4 problem areas were the focal points of the working sessions, 6-week IPT-G focused only on 1 problem area in its working sessions (sessions 2-6), while briefly educating participants of other problem areas that could act as depression problem areas in its psychoeducation session (session 1). The shortened 6-week protocol is provided in the eMethods in Supplement 2.

Each session was led by a facilitator employed by the nonprofit organization StrongMinds International. A total of 4 facilitators, 2 randomized into each district, were assigned to the trial. The facilitators, primarily local individuals with a personal interest in the role—some of whom may have previously participated in IPT-G—underwent a comprehensive 5-day training program. This included interactive workshops, detailed study materials outlining the principles of the counseling intervention, and ongoing support to ensure adherence to the study protocols. The study additionally employed a clinical psychologist (P.B.) to offer weekly supervision to facilitators and ensure fidelity to the protocol. Each facilitator implemented both types of interventions to control for individual characteristics of the facilitators as an extraneous variable.

### Outcomes

Outcome assessors were employed separately from therapists and were masked to study arm. The primary outcome was depression symptom reduction at 3 months after therapy, measured using the PHQ-9, a 9-item instrument covering the *DSM-IV* depressive disorder criteria that is routinely used for IPT-G eligibility and monitoring in Uganda^26,27,28^ and elsewhere in African.^29,30,31,32,33,34,35^ A score of at least 10 corresponds well to a probable diagnosis of depression^25^ with high sensitivity, specificity, and reproducibility across settings.^36^ In a recent systematic review, psychometric properties of the 10-point cutoff in Uganda included a sensitivity of 92% and specificity of 89%.^11^

Secondary outcomes were measured immediately after therapy and at 3-month follow-up. Tools used included the WHO Disability Assessment Schedule 2.0 (WHODAS 2.0), a 12-item questionnaire regarding the difficulty of carrying out activities in the 6 domains of cognition, mobility, self-care, getting along with people, life activities, and participation,^37^ and the WHO Quality of Life–BREF (WHOQOL-BREF), a 26-question abbreviated version of the WHOQOL-100^38,39,40,41^ covering quality of life in 4 domains—physiological, psychological, social, and environmental—plus 2 questions regarding overall quality of life and health.^41^ Details on measures, scoring, and data management are provided in the eMethods in Supplement 2.

### Statistical Analysis

We estimated changes in depression symptom scores (PHQ-9), disability scores (WHODAS), and quality-of-life domain scores (WHOQOL) as continuous outcomes at the individual level, using a linear mixed-effects regression model while adjusting for clustering of participants within the therapy groups. We tested noninferiority of the primary outcome (PHQ-9 score reduction) using the Satterthwaite method.^42,43^ Secondary outcomes were also analyzed by noninferiority testing. We estimated binary outcomes (≥5-point decrease in PHQ-9 score indicating clinically meaningful symptom reduction, ≥10-point decrease in PHQ-9 score indicating large symptom reduction, and ≥50% reduction in PHQ-9 scores) as odds ratios (ORs) using logistic regression. As a sensitivity analysis, we used multiple imputations to handle missing data. To account for multiple comparisons, *P* values for secondary outcomes were adjusted using Bonferroni corrections. We prespecified a statistical significance threshold of 2-sided *P* ≤ .05. Analyses were performed using the lmerTest package in R, version 4.3.2 (R Foundation). Data are available in eTable 7 in Supplement 3.

## Results

### Baseline Characteristics

Participants were first contacted for recruitment on July 29, 2022, and data were accrued from October 31, 2022, until March 24, 2023. A total of 747 individuals were contacted and prescreened, of whom 560 were assessed for eligibility and 328 were eligible and randomized to receive either 6-week problem area–concordant IPT-G or 8-week problem area–discordant IPT-G (Figure) and grouped into a total of 36 therapy groups (18 per arm) with a median of 9 participants per group (IQR, 8-11 for 8-week and 8-10 for 6-week IPT-G arms). The primary reason for ineligibility was PHQ-9 score of less than 10, indicating less-than-moderate depression symptoms. Baseline characteristics were similar between participants enrolled in each of the 2 arms (Table 1). Participants were predominantly female (303 [92.4%] vs 25 [7.6%] male), with a mean (SD) age of 42.3 (15.2) years. Educational attainment ranged from 71 participants (21.6%) reporting having no education to 49 (14.9%) reporting secondary or a higher level of education. A total of 92 participants (28.0%) were married, 107 (32.6%) were cohabiting with a partner, 50 (15.2%) were separated from their partner, and 68 (20.7%) were widowed.

**Figure.**
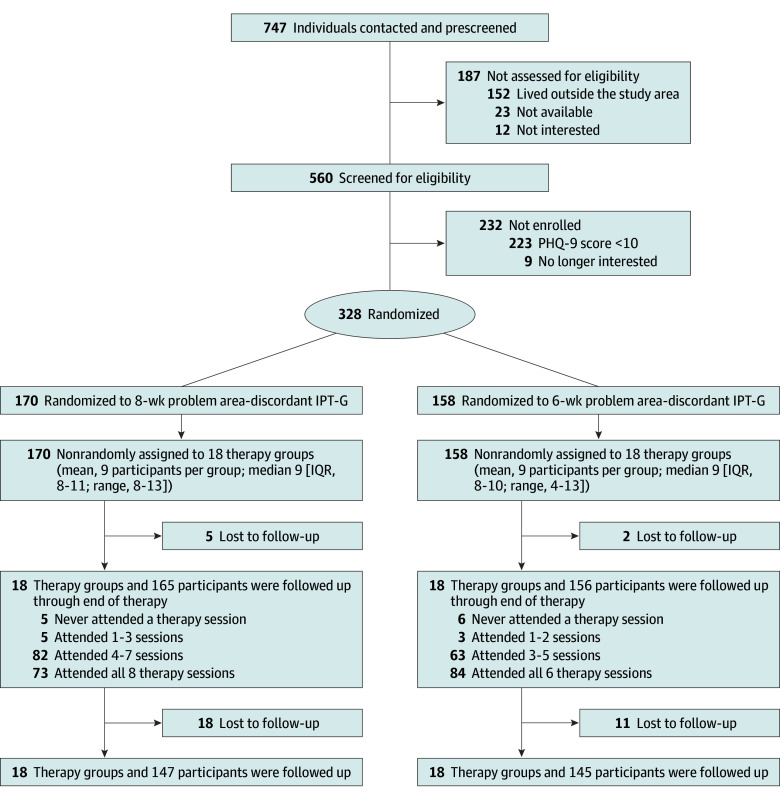
CONSORT Flow Diagram for Participant Recruitment, Screening, Randomization, and Study Retention IPT-G indicates interpersonal group therapy; PHQ-9, 9-item Patient Health Questionnaire.

**Table 1.** Baseline Characteristics of Enrolled Participants by Study Arm

Baseline characteristic	Treatment arm	
	8-wk Problem area–discordant	6-wk Problem area–concordant
All participants, No. (%) (n = 328)	170 (51.8)	158 (48.2)
Age, mean (SD), y	42.3 (16.8)	42.2 (13.3)
Gender, No. (%)		
Female	159 (93.5)	144 (91.1)
Male	11 (6.5)	14 (8.9)
Marital status, No. (%)		
Single	4 (2.4)	7 (4.4)
Cohabiting	59 (34.7)	48 (30.4)
Married	49 (28.8)	43 (27.2)
Separated	21 (12.4)	29 (18.4)
Widowed	37 (21.8)	31 (19.6)
Educational level, No. (%)		
Did not attend school	35 (20.6)	36 (22.8)
Lower primary	46 (27.1)	37 (23.4)
Upper primary	62 (36.5)	63 (39.9)
Secondary or higher	27 (15.9)	22 (13.9)
No. of children, No. (%)		
None	9 (5.3)	4 (2.5)
1	16 (9.4)	6 (3.8)
2-4	36 (21.2)	42 (26.6)
≥5	109 (64.1)	106 (67.1)
District, No. (%)		
Buikwe	82 (48.2)	82 (51.9)
Kayunga	88 (51.8)	76 (48.1)
Baseline PHQ-9 score, mean (SD)^a^	16.80 (4.35)	17.59 (4.33)
Baseline depression severity, No. (%)		
Mild	0	0
Moderate	65 (38.2)	49 (31.0)
Moderately severe	59 (34.7)	56 (35.4)
Severe	46 (27.1)	53 (33.5)
Baseline WHODAS 2.0 score, mean (SD)^b^	21.2 (8.5)	21.1 (7.8)
Baseline WHOQOL-BREF score, mean (SD)^c^	46.7 (8.7)	47.4 (8.8)
Baseline WHOQOL-BREF domain scores, mean (SD)^c^		
Physiological	51.0 (18.4)	51.8 (18.9)
Psychological	53.0 (15.5)	55.9 (15.6)
Social	40.5 (22.1)	41.5 (23.2)
Environmental	47.4 (16.7)	47.3 (17.4)
Problem areas, No. (%)^d^		
Disagreement	61 (35.9)	56 (35.4)
Grief	27 (15.9)	29 (18.4)
Isolation	11 (6.5)	7 (4.4)
Life change	142 (83.5)	141 (89.2)
No. of problem areas, No. (%)		
1	101 (59.4)	82 (51.9)
≥2	69 (40.6)	76 (48.1)

Abbreviations: PHQ-9, 9-item Patient Health Questionnaire; WHODAS 2.0, World Health Organization Disability Assessment Schedule; WHOQOL-BREF, World Health Organization Quality of Life Questionnaire.

^a^ Scores range from 0 to 27, with scores of 10 or greater indicating probable depression.

^b^ Scores range from 12 to 60, with higher scores indicating greater disability.

^c^ Scores range from 0 to 100, with higher scores indicating higher quality of life.

^d^ A total of 145 patients specified more than 1 area.

Similar numbers of participants were categorized as having different depression symptom severities: 114 (34.8%) moderate (PHQ-9 score 10-14), 115 (35.1%) moderately severe (PHQ-9 score 15-19), and 99 (30.2%) severe (PHQ-9 score ≥20). Mean (SD) baseline PHQ-9 score was 17.18 (4.35). Mean (SD) baseline disability score was 21.1 (8.2), indicating severe morbidity.^44^ Mean (SD) baseline total quality of life score was 47.1 (8.7), indicating substantially lower quality of life compared with the general population in central Uganda.^45^ The most commonly reported problem area for depression was life change (283 [86.3%]), followed by disagreement (117 [35.7%]); 145 participants (44.2%) reported multiple depression problem areas.

### Retention, Attendance, and Change in Depression

Study retention was high, with 321 participants (97.9%) retained for the outcome assessment at the end of therapy and 292 (89.0%) retained for follow-up assessment at 3 months post therapy (Figure). Retention was similar by arm, and balance of baseline characteristics across arms was maintained after attrition (eTables 1 and 6 in Supplement 2). In the 6-week problem area–concordant arm, mean (SD) PHQ-9 scores were 17.59 (4.33) at baseline, 2.4 (2.7) at the end of therapy, and 4.4 (4.5) at 3 months post therapy. Mean (SD) decline in PHQ-9 score was 15.2 (5.1) from baseline to the end of therapy and 13.3 (6.1) points from baseline to 3 months post therapy. In the 8-week problem area–discordant arm, mean (SD) PHQ-9 scores were 16.80 (4.35) at baseline, 3.5 (3.7) at the end of therapy, and 5.3 (4.9) at 3 months post therapy. Mean (SD) decline in PHQ-9 score was 13.3 (5.3) from baseline to the end of therapy and 11.4 (6.7) from baseline to 3 months post therapy.

### Primary Outcomes

We found 6-week problem area–concordant therapy to be noninferior to 8-week problem area–discordant therapy. The noninferiority margin of 1 point on the PHQ-9 was achieved at end of therapy (*t*_318.92_ = −4.96; *P* < .001) and 3 months post therapy (*t*_288.15_ = −3.97; *P* < .001).

Differences in PHQ-9 score improvements were statistically significant between arms. Compared with the 8-week problem area–discordant arm, PHQ-9 scores in the 6-week problem area–concordant arm decreased by an additional 1.86 (95% CI, 0.74-3.00) points (*P* = .001) from baseline to end of therapy and 1.98 (95% CI, 0.60-3.36) points (*P* = .005) from baseline to 3 months post therapy, with mean (SD) PHQ-9 scores of 11.35 (6.71) and 13.34 (6.71) for the 6-week and 8-week arms, respectively. The intraclass correlation coefficient for PHQ-9 scores was 0.111.

### Secondary Outcomes

Disability showed statistically significant improvements from baseline in both arms. The time × arm improvement in disability scores was −2.70 (95% CI, −4.44 to −0.95) points on the WHODAS from baseline to end of therapy (*P* = .02). Changes in WHODAS at 3-month were not significant, nor were changes in the 4 WHOQOL domains. The intraclass correlations for WHODAS and WHOQOL scores were 0.310 and 0.308, respectively (Table 2).

**Table 2.** Secondary Outcomes

Disability and quality-of-life score changes^a^	End of therapy		3 mo Post therapy	
	β estimate (95% CI)	*P* value^b^	β estimate (95% CI)	*P* value^b^
WHODAS,^c^ time × arm (6-wk vs 8-wk arm after treatment)	−2.70 (−4.44 to −0.95)	.02	−0.68 (−2.69 to 1.34)	>.99
WHOQOL physiological domain, time × arm (6-wk vs 8-wk arm after treatment)	5.66 (0.87 to 10.45)	.11	0.77 (−5.30 to 6.85)	>.99
WHOQOL psychological domain, time × arm (6-wk vs 8-wk arm after treatment)	1.07 (−3.21 to 5.34)	>.99	−0.04 (−4.72 to 4.65)	>.99
WHOQOL social domain, time × arm (6-wk vs 8-wk arm after treatment)	2.13 (−3.25 to 7.51)	>.99	−0.89 (−7.07 to 5.29)	.99
WHOQOL environmental domain, time × arm (6-wk vs 8-wk arm after treatment)	4.71 (0.48 to 8.95)	.15	3.63 (−1.40 to 8.66)	.79

Abbreviations: WHODAS, World Health Organization Disability Assessment Schedule; WHOQOL, World Health Organization Quality of Life Questionnaire.

^a^ Results are reported as fixed effects in a linear mixed effects model, with β coefficients in units of score change from baseline.

^b^ Bonferroni adjusted.

^c^ Adjusted intraclass correlation coefficient at baseline for WHODAS score was 0.310 and for total WHOQOL score was 0.308.

Depression symptom reduction was also noninferior at the end of therapy assessment. Additionally, at end of therapy, the proportion of participants with large (≥10-point) PHQ-9 score improvement was significantly larger in the 6-week problem area–concordant arm (OR, 2.79; 95% CI, 1.40-5.56). There was no statistically significant difference between arms in the other 2 PHQ-9 binary response measures of clinically meaningful (≥5 points or ≥50%) score improvement. There were no statistically significant differences in binary response measures between arms at 3-month follow-up (eTable 2 in Supplement 2). Pooled results from the sensitivity analysis for primary and secondary outcomes indicated that the findings were not sensitive to the handling of missing data (eTables 3-5 in Supplement 2).

### Adverse Events

No study-related adverse events were recorded. Two serious adverse events occurred during the study period, both in the 8-week problem area–discordant arm. Neither event was determined to be study related.

## Discussion

This randomized clinical trial is the first study, to our knowledge, to test an adapted 6-week problem area–concordant IPT-G model in which clients with a common problem area for depression were grouped together and treated with 25% fewer sessions compared with 8-week problem area–discordant IPT-G. The study met all primary and some secondary end points. Six-week problem area–concordant IPT-G was noninferior to 8-week problem area–discordant IPT-G in reducing depression symptoms. Symptoms were reduced by a mean of 1.86 additional points on the PHQ-9, and the difference was maintained after 3 months. Self-reported disability and quality of life improved substantially in both arms. Participants receiving 6-week problem area–concordant IPT-G reported significantly less disability immediately after therapy, though these differences were not statistically significant after 3 months.

Our findings indicate that 6-week problem area–concordant IPT-G is an effective alternative to the current 8-week problem area–discordant IPT-G in treating depression symptoms. Improving the efficiency and effectiveness of group-based psychotherapy is a priority for resource-constrained settings such as Uganda, home to millions of people with depression. Interpersonal counseling, a briefer version of IPT, has been implemented in some resource-constrained settings as a less intensive treatment alternative. However, IPT-G has an established workforce and evidence base in Uganda, making it a more readily scalable option for our study. StrongMinds International, Uganda’s largest mental health nongovernmental organization, treated more than 150 000 clients in 2023. For this organization alone, switching to 6-week problem area–concordant therapy would have allowed more than 35 000 additional clients to be treated with the available cadre of mental health professionals. Approximately 300 000 visits would have been avoided, reducing the burden of therapy attendance and potentially improving treatment accessibility for individuals with circumstances preventing them from attending numerous appointments. Evidence from this study can be used to both expand and improve depression treatment.

Our study adds to prior research demonstrating that shortened psychotherapy can be effective for treating depression. Most notably, Shapiro et al^46^ found that shorter psychotherapies could achieve comparable outcomes to longer treatments, particularly when tailored to patient needs and depression severity. In a systematic review of 15 randomized clinical trials, Nieuwsma et al^47^ found that 6 to 8 sessions of cognitive behavioral therapy was effective compared with no therapy, a finding reinforced by more recent trials.^48,49,50^ However, most of these studies used control conditions such as a waitlist or attention control, rather than directly comparing longer vs shorter therapy using a noninferiority design as reported herein. A noninferiority study design was selected to determine whether the investigated shortening of IPT-G maintains effectiveness while offering potential advantages, such as reduced demands of participant and therapist time, which is important especially in settings where the need for mental health services far exceeds availability of therapists. This approach aligns with ethical considerations in study design and facilitates the evaluation of treatments where existing therapies are effective but may have limitations.^51,52^ Previous literature, such as the study by Wilfley et al^53^ on structured IPT groups and postpartum depression, demonstrated the effectiveness of grouping clients with common depression problem areas to enhance therapeutic outcomes.^54^ However, our study is also the first, to our knowledge, to specifically compare 6-week problem area–concordant groups with 8-week problem area–discordant groups. This approach, in which we found 6-week IPT-G to be noninferior to 8-week IPT-G, increases the scalability of the intervention by using the same available staff resources to treat more people.

Multiple mechanisms could have contributed to the effectiveness of the 6-week arm compared with the 8-week arm. Group-based therapy includes a social support component in which clients empathize with one another and suggest solutions to reducing depression symptoms. Clients often make plans for continued social support even after therapy, such as exchanging telephone numbers, scheduling social visits, and checking in if they do not hear from one another.^54^ Grouping clients with a common problem area may help customize and strengthen peer support. Additionally, problem area–concordant IPT-G may increase the proportion of therapy content that clients find relevant to their circumstances, facilitating engagement, learning, and long-term retention of concepts. Focusing primarily on one problem area may reduce cognitive load for clients, for which they are at increased risk due to circumstances associated with depression (eg, poverty).^20^ Finally, shortened therapy may have been perceived as more feasible and manageable by clients, further facilitating engagement. However, given the nature of our study design, the interpretability of the specific mechanisms underlying the findings is limited. Future research could aid in clarifying the main mechanisms by which the 6-week arm was noninferior to the 8-week arm.

### Limitations

Our study has several important limitations. First, we conducted it in central Uganda, and all study sites had commonalities, including language (Luganda), geotype (rural), socioeconomic status (low income), livelihoods (smallholder farming), and other factors. Generalizability to populations underrepresented in this study will require additional research. Second, multiple testing was conducted in our analyses, increasing the risk of type I errors; however, we used Bonferroni corrections to adjust the *P* values of secondary outcomes. Third, outcomes were measured after equal amounts of time relative to the end of therapy, which were staggered by 2 weeks relative to the start of therapy, given the 2-week difference in therapy duration across the 2 arms. This choice was made to capture the most immediate effects of treatment and possible waning of effects after treatment ends. Because our primary outcome was measured 3 months post therapy, this choice was unlikely to have made a large difference in the study results but could merit further investigation. Fourth, due to logistical barriers, trial registration occurred after all participants had been treated. While this delay did not involve changes to the study design or protocol, it should still be considered when interpreting the study’s findings. Finally, currently available follow-up data are limited to 3 months after treatment. While IPT-G has shown durable benefits compared with no treatment,^19^ focusing on a single problem area may leave clients less prepared for future depression relapses arising from new life experiences, compared with standard of care. However, IPT-G principles are designed to be generalized to new experiences, and clients are supported in developing action plans should symptoms relapse regardless of IPT-G model. Moreover, even if shortened problem area–concordant IPT-G were less durable, its comparable efficacy may still make it a preferred option for treating active depression. Future research is needed to compare the durability of problem area–concordant vs problem area–discordant IPT-G for remaining depression free.

## Conclusions

In this randomized clinical trial, 6-week problem area–concordant IPT-G was noninferior to 8-week problem area–discordant IPT-G for reducing depression symptoms. Our findings of noninferiority can directly inform IPT-G delivery to enhance efficiency and scalability and thereby reduce the depression treatment gap in resource-constrained settings.

## Notes

Supplement 1.
Trial Protocol

Supplement 2. **eMethods.** Sample Size, Setting and Recruitment, Treatment, Outcomes, and Data Management **eTable 1.** Baseline Characteristics of Study Participants Retained to End of Therapy and 3-Month Posttherapy Follow-Up **eTable 2.** Secondary Outcomes of the Effect of Shortened, Trigger-Concordant Interpersonal Group Psychotherapy on Binary Treatment Response Measures (PHQ-9) **eTable 3.** Pooled Results From Multiple Imputation Analysis of the Effect of Shortened, Problem Area–Concordant Interpersonal Group Psychotherapy on Depression Scores (PHQ-9) at End of Therapy and 3 Months Post Therapy **eTable 4.** Pooled Results From Multiple Imputation Analysis of the Effect of Shortened, Problem Area–Concordant Interpersonal Group Psychotherapy on Self-Reported Disability, and Self-Reported Quality of Life in the Domains of Physiological Health, Psychological Health, Social Relationships, and Environment at End of Therapy and at 3 Months Post Therapy **eTable 5.** Pooled Results From Multiple Imputation Analysis of the Effect of Shortened, Problem Area–Concordant Interpersonal Group Psychotherapy on Binary Treatment Response Measures (PHQ-9) **eTable 6.** Baseline Characteristics of Study Participants Stratified by Retention Status

Supplement 3.
eTable 7.

Supplement 4.
Data Sharing Statement
