# Supplementary material for: Six-Week Problem Area–Concordant vs 8-Week Problem Area–Discordant Group Interpersonal Psychotherapy: A Randomized Clinical Trial
Source: JAMA Netw Open. 2025 Apr 16;8(4):e255242. doi: 10.1001/jamanetworkopen.2025.5242 (PMC12004200; doi:10.1001/jamanetworkopen.2025.5242)
Supplement: Supplement 1. — Trial Protocol [file jamanetwopen-e255242-s001.pdf]

Interpersonal Psychotherapy for Groups adapted for the Ugandan Population: A randomized controlled trial of six sessions versus eight sessions of trigger concordant and trigger discordant therapy

Date: 19th September 2022

Principal Investigator

Rosco Kasujja, PhD – Innovations Director, StrongMinds Global
Senior Lecturer, Makerere University School of Psychology

Co-investigators

Anna Bershteyn, (Ph.D.) ---Assistant Professor of Population Health, New York University
Katia M Peterson (Ph.D.) ---Research & Evaluation Consultant
Christina Angela Ntulo--- Public Health Specialist/Country Director, StrongMinds-Uganda
Vincent Mujune---Head of Programs, StrongMinds Uganda
Charlotte Oloya---Head, Communications, Partnerships & Advocacy-StrongMinds Uganda

Research Coordinator

Peter Birungi -MSc – Research Coordinator.

Programme Team

Costella M. Tindyebwa----Head, Monitoring, Evaluation, & Learning-StrongMinds Uganda
Charlotte Oloya---Head, Communications, Partnerships & Advocacy-StrongMinds Uganda
Leah Turner---Research and Evaluation Consultant

Funder

Internal Funding by StrongMinds Global

A study protocol for review by Makerere School of Health Sciences Research Ethics Committee (MAKSHS-REC), Makerere University

Introduction

Depression is estimated to affect over three hundred million (300,000,000) people of all ages worldwide. The World Health Organization (WHO) describes it as the leading cause of ill health and disability in terms of total years lost due to disease in the world (Health data, 2021). According to The Diagnostic and Statistical Manual of Mental Disorders, Fifth Edition (DSM-5) depression is a mood disorder identified by a loss of interest or pleasure in daily activities for more than two weeks by an individual. It is characterized by increased sadness, emptiness, helplessness, worthlessness, and thoughts of being unlovable. Depression has serious personal, interpersonal and societal consequences that affect one's social life and career as one losses their sense of enjoyment of everyday activities, are endlessly unhappy, have unwarranted ideas of guilt, have difficulty concentrating, and experience appetite disturbance and sleep problems (APA, 2013).

Uganda has a population of over 47.6 million people (World Bank, 2022) and is considered a low-income country, with a gross national income is below **US$**1,026 and most of the population living below the poverty threshold (UBOS, 2020). Women experience economic disparities more than men, and struggle to effectively carry out their responsibilities without intimidation and harassment. They are disproportionately susceptible to feeling frustrated, hopeless, and helpless, and to feeling unwanted and rejected, which can predispose them to depression (Fischer, 2016). WHO described depression in women as a factor contributing to poor growth in children; a mother's mental health influences the growth of their children with effects not only affecting the current generations but the next generations especially in the low-income countries like Uganda (Nabunya et al. 2020).

Depression is highly prevalent in rural Ugandan communities. According to Kaggwa et al. (2021) rural women are highly prevalent to depression with their study reporting over 65 percent of women in rural Uganda had depressive symptoms. IPT-G has been used successfully in these regions following the IPT-G-U adapted manual (Lewandowski et al., 2016), but due to lack of providers and financing for mental health care, most women and girls are three times more likely than boys to experience depressive disorders (Nabunya et al. 2020) and go untreated.

StrongMinds Background

Strong Minds is a social enterprise founded in 2013 that empowers impoverished African women by treating depression at scale and enables these women and their families to lead more healthy, productive, and satisfying lives. Strong Minds treats depression through a community-based talk therapy model known as Interpersonal Psychotherapy for Groups

(IPT-G), in which therapy groups are led by a facilitator over a period of 8 weeks to help members identify the root causes and triggers of their depression and formulate strategies to overcome those triggers. Since depression is episodic and recurrent throughout most people's lives, these newly acquired skills have both immediate and long-term preventive impacts for the sufferer.

Results from StrongMinds have been like outcomes measured in randomized controlled trials of IPT-G. Participants who complete our talk therapy groups not only resolve their current depressive state — they also learn coping strategies to help prevent future depressive episodes. Roughly 75% of the groups elect to continue to meet after formal sessions conclude.

Problem statement

IPT-G has been widely used and tested in Uganda by various organizations to treat depression which makes it one of the best treatments for depression. However, many practitioners have noted that participants become either demotivated or unavailable to attend sessions after attending the fourth and fifth sessions. Practitioners have questioned the importance of delivering the 8-16 weekly sessions model as recommended by the world health organization (WHO), considering that the time spent offering the later, poorly attended sessions could be redirected toward launching additional treatment groups to reach more patients in need.

Additionally, providers have suggested that sitting in a group session with individuals whose depression was caused by triggers different from others can be discomforting and demotivating. It is unknown whether people with depression get better after a few sessions irrespective of having the same triggers or they just get bored, disengaged and ultimately demotivated due to differences in the triggers. Providers hypothesize that treatment groups in which all participants have the same depression trigger (i.e., "trigger-concordant" groups) may be more effective than standard-of-care groups in which participants have a mix of depression triggers (i.e., "trigger-discordant" groups).

This study aims at testing the efficacy of an adapted short version of IPT-G (6 trigger-concordant sessions) compared to the current standard of care for IPT-G at StrongMinds (8 trigger-discordant weekly sessions) and a waiting list control group.

Objectives:

The objectives of the study include the following:

Main Objective: To compare the effectiveness of 6 trigger-concordant IPT-G to 8 trigger-discordant IPT-G sessions on reducing depressive symptomatology among individuals in Uganda. Both therapies will be compared to a control group (delayed treatment).

Specific Objectives

1. To compare the changes in depressive symptoms between participants who receive 6 trigger-concordant weekly IPT-G sessions versus 8 trigger-discordant weekly IPT-G sessions.
2. To examine the difference in the changes in functionality between participants who receive 6 trigger-concordant weekly IPT-G sessions versus 8 trigger-discordant weekly IPT-G sessions
3. To assess the changes in the quality of life between participants who receive 6-weekly IPT-G sessions focusing on trigger concordance to those who receive 8-weekly IPT-G sessions focusing on trigger discordance.

IPT-G Interventions (Treatment as Usual= TAU)

The 8 sessions of therapy are broken into three phases, each with distinct objectives:

1. Session 1 (Initial Phase)

   This phase focuses on creating initial bonds between group members and building rapport with one another, so participants feel comfortable sharing personal information and discussing the reasons for their depression. Participants' goals and specifically problem areas that triggered depressive symptoms for each participant are reviewed. At this phase, members get to know one another as they embrace for the middle phase which is also known as the working phase.

2. Session 2-7 (Middle Phase)

   This phase ensures that all members are actively engaged and helping each other by making suggestions regarding one another's problems. This is also the phase where members are educated about the symptoms and common triggers of depression. Priority is given to working toward everyone's goals by brainstorming to raise as many solutions as possible to problems. Homework is key at this stage as weekly events that tend to affect the mood are analyzed every week.

3. Session 8 (Termination Phase)
   This phase focuses on preparing members to end formal sessions. Members are reminded to continually identify their own triggers of depression in the future and what they should do to respond. Individual action plans are created and reviewed.

Description and delivery of the trial intervention (IPT-G six sessions)

The adapted IPT-G model will focus on one common IPT trigger for depression throughout treatment (six sessions). All groups will comprise participants with one common trigger for depression with a focus on treating their depressive symptomatology over six sessions. A brief description of the six sessions intervention is provided below.

Session one (Initial Phase)

This is an introductory session and will aim at providing introduction for the group participant who will be meeting each other for the first time. Psychoeducation about depression and how the group will work will be offered at this stage. The facilitator will also ensure that the participants work out some of the group rules and clarify treatment goals. This group sets the stage by ensuring that all participants realize that they are depressed and that their symptoms resulted from four IPT triggers.

Sessions two to five (Middle Phase)

This is also known as the working phase. Each participant (in a group setting) will work to improve their symptoms following the guidance of a facilitator who will use IPT strategies that are specific to the trigger presented by group participants, and techniques including decision analysis, communication analysis, role-plays, administering homework, reviewing symptoms of depression, and linking mood to events and events to mood each week throughout the four weeks.

Session Six (Termination)

This session focuses on reviewing goals of the client and checking to verify whether the participants have improved after the treatment. Focus is on how the participants will cope after the group intervention. Information regarding acknowledging and dealing with relapse and reflection on the actual group experience is done. A thorough review of what has been done throughout the six session and how participants can use it to their benefit in their day-to-day life is carried out in this last session. Information on where to seek support in case of relapse is also provided during this last session.

Role of Mental Health Facilitators (MHFs)

At the start of the RCT, the MHFs delivering the intervention will receive a specific training, coordinated by the researcher, regarding the nature of the intervention as described above. This training will be conducted in the form of interactive workshops, conducted by the investigators, and will include demonstrations and role play. The training will include specific counselling techniques required in this study. This will include aspects such as introduction, building up a rapport, listening to the participant, reflecting, helping the person himself/herself identify the problem or trigger that led up to depression among others. The need for maintenance of privacy and confidentiality will be stressed. There will be also a focus and discussion on limits and boundaries and identification of risks, as well as when and how to refer for further help. The MHFs will also be given a structured manual which outlines the principles of the counselling intervention, and they will be encouraged to use this as a guide when delivering the intervention.

During the facilitators' training, all attempts will be made to build confidence and provide a positive background towards carrying out this role in the ward. Possible challenges the facilitators may face, such as time constraints, and other issues will be looked for and discussed during these workshops. Facilitators will be encouraged to carry out practice sessions of the intervention for a cycle before the trial begins to boost confidence and fidelity to the intervention.

There will be an ongoing contact between the MHFs involved in the study and the researcher to monitor for any difficulties during the implementation of the intervention. Before starting the intervention, the investigators will provide a description about the background and rationale of doing this study to other health staff who will be working at the study site to enlist their support.

All participants (intervention, and control group) if deemed at risk because of attending the study sessions or due to the delayed treatment will receive psychiatric referral, as deemed required by the facilitators (which is the usual practice) irrespective of the case/control status of the participant.

Delivery of the intervention and the safety of participants

The intervention will be delivered by four designated trained mental health facilitators (MHF) who have an interest in this new role and are willing to deliver the intervention. The details of the facilitators training have been described previously. It is not possible to use a single

MHF in two different districts. The study will have four facilitators in total who will be trained to use standardized protocols for 6 and 8 weeks IPT models. Two facilitators will be randomly assigned to each district and will be expected to each implement both 6 and 8 weeks of IPT. This guarantees that individual characteristics of the MHFs as an extraneous variable will to some extent be controlled. In addition, weekly supervision will be offered to all the MHFs to boost fidelity to the approach. Additionally, MHFs will be offered training sessions before the actual trial to ensure that sessions guides are understood and implemented the same way.

The distinction between mild and moderate depression is only defined by the PHQ-9 screening tool. Therapeutic approaches, including IPT-G, do not change based on the 'severity' of the depression as defined by the screening tool. There are no guidelines or best practices that govern a change in the therapeutic curriculum to specifically respond to an individual based on the intensity of their depression level. Moreover, there is no evidence that this sort of tailored therapeutic response is necessary to protect the safety of participants unless those individuals are determined to be suicidal and/or at risk of harming themselves or others. As is the standard practice with most depression therapeutic curricula, all individuals with depression, regardless of the severity of their symptoms, will be treated the same, unless they exhibit imminent risk of suicide, of harming themselves or of harming others. A psychiatrist at Butabika National Referral Hospital has been included in this study and will be on standby to manage emergencies that might come up during the trial.

Study Design

The study is designed as a randomized controlled trial with the main effects being whether the new 6-weekly IPT-G model (trigger concordant), and the 8-weekly IPT-G (trigger discordant) model differ in terms of effectiveness from a control group and each other. Participants will be selected following a single-case cluster-randomized controlled trial. The approaches to be used for data collection and analysis will be mixed (qualitative and quantitative) methods. All tools will be locally adapted as StrongMinds has been using them for some time. Three sets of participants will be enrolled in the study; Treatment as usual (TAU) will be 8-weekly sessions of IPT-G as administered by StrongMinds mental health facilitators, 6-weekly IPT-G sessions will be the intervention group and a control group that will be on a waiting list to receive one of the treatment options after the conclusion of the study.

Setting

The study will be conducted among depressed participants in Jinja and Kayunga districts. These districts are geographically and culturally similar. Jinja as eleven sub-counties while Kayunga has nine sub-counties. Two sub-counties from both Jinja and Kayunga will be randomly selected and assigned either as the control or intervention group. The study will ensure that each district gets one intervention and one control sub-county. Equal numbers of participants will be selected from each sub-county and randomly assigned to either the control or intervention group.

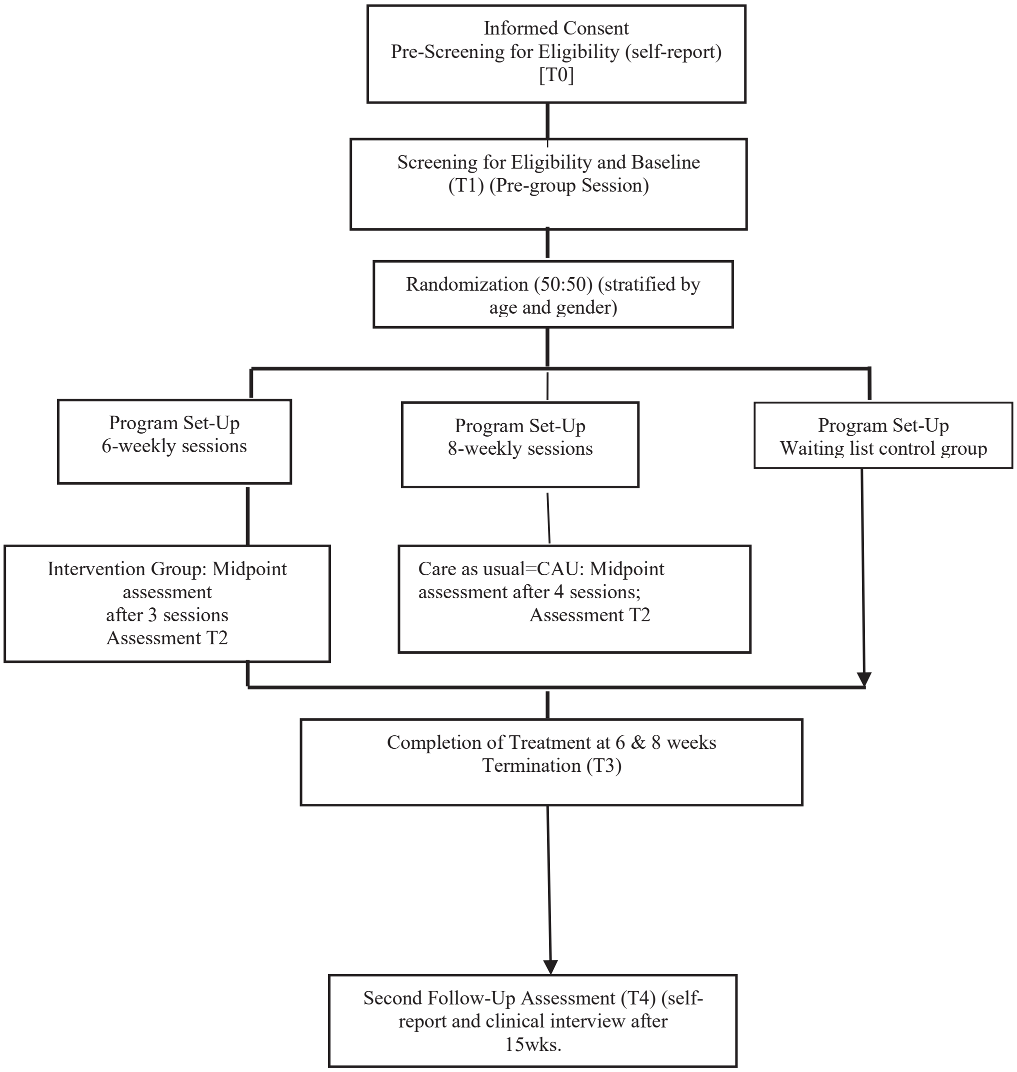

Participants

Individuals who complete the standard StrongMinds intake interview and screen positive for depression symptoms will be offered information about the research trial before proceeding

to conduct a pre-group session and final enrollment into the trial. All participants (both males and females) will be screened using the PHQ9. Those who score 10 and above on the PHQ-9 and share at least two IPT problem areas will qualify for enrollment into the study if they offer consent. Recruitment will be pursued until the desired sample size is realized at both sites.

Sample size selection

The study will employ block randomization within each stratum by ensuring that all participants can be assigned to either six or eight sessions (single or different triggers). Randomization will also consider the age and gender of a participant. A total of 300 participants will be recruited to participate in the study following recruitment interviews with participants going through pre-group sessions. The sample size is based on a previous effect size calculation of 8,800 StrongMinds' client scores. The calculation determined the sample size required to detect a one and two-point difference of PHQ-9 scores as a continuous outcome. To recruit the fewest number of study participants, while still achieving the goals of the study, the study team has opted to recruit the number of participants required to detect a two-point difference. Accounting for 50% attrition, based on previous StrongMinds' studies and program findings, the required sample size is 150 participants for each study arm (300 total).

Effect size (PHQ-9 score as a continuous outcome	Sample size required per group
2-point difference on depression score pre vs post	99
1-point difference on depression score pre vs post	387

Notes: The above calculation is based on the assumptions of (1) $\alpha = 0.05$ (two-tailed test, repeated measure design), (2) $\beta = 0.20$ (power=0.80), 3) standard deviation of depression outcome = 5.00 (according to the previous data), and 4) within subject correlation on outcome depression measure = 0.02 (based on the previous data).

Using Redcap software, 150 participants will be randomly assigned to the 6-session model with trigger concordance and the 150 patients assigned to the 8-session treatment as usual. The sample is set at a 95% significance and 80% power. All the participants will be assessed to have 2-3 common triggers before randomization into the various intervention arms. Once treatment begins the facilitators for the various arms will either; a) **for the concordant group**, choose and focus on a singular shared trigger while b) f**or the discordant group**, the facilitator will focus on all the different triggers during the session.

Inclusion criteria

Persons aged 18 years and over screened using the PHQ-9 and reported to have depression (cut off score of 10 and above on the PHQ-9), with two or more triggers of

depression. Participants with at least two similar triggers out of the four IPT triggers to depressions will be included in the study. Participants must be fluent in either English or Luganda and be able to consent to participation.

Exclusion criteria

Persons who state or carry reports indicating a previous diagnosis of intellectual disability, psychiatric disorder other than depression, or dementia, and those who are physically too unwell to participate in the interview will be excluded. Participants who meet the above inclusion criteria but have impairments that hinder engagement with the research procedures for any reasons (is deaf or hard of hearing, speech, and visual impairment with no aids to see, unable to give consent will be excluded. Those who are not fluent in either spoken English or Luganda and under the age of 18 will also be excluded from the study. Due to the study location where it is expected that all residents speak Luganda, any participants who confesses that they cannot hear nor understand Luganda will be excluded from the study.

Potential participants will receive a participant study information sheet or a clear description of the study by any of the research assistants and any of their questions will be answered. Written consent will be sought. Only those who give written informed consent will be recruited to the study.

Recruitment

A team of Mental health facilitators will provide a talk about depression in the communities and encourage individuals to come for screening. Participants who meet the inclusion criteria as described above, who have given written and/or verbal informed consent, will be screened for depression using the PHQ-9. Screening will be at two levels. First the PHQ-2 will be used to screen for depressed mood and loss of interest in everyday activities. Those who score ≥3 will be regarded as positive for depression and will have to undergo further screening using the full PHQ-9. Upon administering the PHQ-9, any individual with a score of ≥ 10 will be randomly assigned to either the control, intervention or waiting list study arm in both Jinja and Kayunga districts. Participants recruited to take part in the study in either the intervention, control or waiting list arms will undergo four levels of assessment, that is, at baseline during a one-on-one screening session with their group facilitator, at mid-point (three and four weeks respectively), termination, and follow-up after three months.

Recruitment and intervention will be conducted over a consecutive two 10-week cycles, or until the required number of participants are enrolled, and the end date of the study will be six months after the baseline intervention.

Variables

The primary dependent variable in this study is the PHQ-9 score change between pre- and post-therapy using the PHQ-9. The dependent variables include treatment type, age, gender, and location. Other outcome variables will include quality of life and functioning.

Measurement

PHQ-9*:* The Patient Health Questionnaire (PHQ-9) is a self-administered version of the PRIME-MD (Primary Care Evaluation of Mental Disorders) diagnostic instrument for common mental disorders. The PHQ-9 is the depression module, which scores each of the 9 DSM-IV criteria as "0" (not at all) to "3" (nearly every day). PHQ-9 scores of 5, 10, 15, and 20 represented mild, moderate, moderately severe, and severe depression, respectively.

To determine the level of depression, the standard Patient Health Questionnaire (PHQ-9) will be used to generate a score. The PHQ-9 is an ordinal scale tool that measures the frequency that cardinal depression symptoms are measured. The PHQ-9 consists of 9 questions, each with response options of 0 (never), 1 (several days), 2 (more than half of the days), and 3 (nearly every day). The PHQ-9 has been widely used in Uganda (Kaggwa et al. 2022) and has been adapted and translated for the Ugandan population in both Luganda and Rutooro (Miller et al. 2021). The checklist will be administered at three time points. That is, before participants start therapy (at pre-group), at midline (after three sessions for 6 weeks group, and after 4 sessions for 8 weeks group) and at termination (at the end of all sessions targeting all participants who attended therapy whether they dropped out or not).

Having a clinically significant score change from baseline will be measured based on computation of the difference between the pre-group PHQ-9 score and the end line PHQ-9 score for each client. Participants found to have a 5 plus score change will be categorized as having a clinically significant score change.

WHO Quality of Life BREF: The World Health Organization Quality of Life (WHOQOL) project was initiated in 1991. The aim was to develop an international cross-culturally comparable quality of life assessment instrument. It assesses the individual's perceptions in the context of their culture and value systems, and their personal goals, standards, and

concerns. The WHOQOL instruments were developed collaboratively in several centers worldwide. The WHOQOL-BREF instrument comprises 26 items, which measure the following broad domains: physical health, psychological health, social relationships, and environment. The WHOQOL-BREF is a shorter version of the original instrument that is more convenient for use in large research studies or clinical trials. It has been widely used and the PI has adapted for use in Uganda amongst Congolese Refugees.

WHO Disability Assessment Schedule (WHODAS 2.0): The World Health Organization Disability Assessment Schedule (WHODAS 2.0) is a generic assessment instrument developed by WHO to provide a standardized method for measuring health and disability across cultures. The WHODAS 2.0 is the current leading measure of disability worldwide (Federici et al. 2016). It was developed from a comprehensive set of International Classification of Functioning, Disability and Health (ICF) items that are sufficiently reliable and sensitive to measure the difference made by a given intervention. The WHODAS 2.0 is a generic health and disability measure that describes the effects of disease on six domains including cognition, mobility, self-care, getting along, life activities, and participation in society. Disability perception is measured on 5-point scale ranging from 1 (no difficulty) to 5 (extreme difficulty or cannot do). This will be achieved by assessing the same individual before and after the intervention. The WHODAS 2.0 was found to be useful for assessing health and disability levels in the general population through surveys and for measuring the clinical effectiveness and productivity gains from interventions. The tool has been widely used in Africa specifically in Congo (Keyser et al. 2022), Ethiopia (Habtamu et al. 2017), and in Uganda (Hamid et al. 2017; Scholten et al. 2011; Vancampfort et al. 2021) among others. In this study we shall use the total score with a higher score indicating greater impairment of health status.

Qualitative data collection

Qualitative data on the perceived benefit of the two study arms will be collected through in-depth interviews from individuals diagnosed with depression. Interviews will be conducted with select clients who will have either completed or not completed treatment. Interviews will be audio-recorded and later transcribed in preparation for data analysis. Debriefing forms will also be completed by the qualitative researcher after each interview to record and reflect on important behaviors, events, and other features of the interview. In addition, process notes on the intervention will be collected and will include time spent on each session, questions raised by participants, and approaches to the mentioned therapy. Data from clients and key

informants will be collected at the end of the intervention with the aim of improving the program. The total number of participants to be targeted during the qualitative interviews will be 20 with 10 from each study arm.

Data management

Any hard copy data collected throughout the study process will be safely stored at StrongMinds Global Office. Participants will be randomly assigned a unique participant identification number which will be used in lieu of names or other identifying information. All de-identified quantitative and qualitative data (translated PDFs) will be shared and harmonized through a secured research data storage (e.g., RedCap). The trial management steering team will have access to the de-identified data via this software (e.g., RedCap). To ensure secure and safe procedures are continuously adhered to, a data management protocol will be developed and adhered to in coordination with each site.

All tablets used by research assistants will be programmed with must-enter variables, range checks, repeat variables, and conditional jumps to improve data quality. Data will be checked daily by a team supervisor for completeness and correctness before being uploaded into an online database managed by the research team.

Statistical Methods and Analysis

1. Analyses of treatment completion: To understand whether there is a differential rate of completion for treatment conditions, a logistic regression will be conducted. Completion will be defined as attending at least (3 or 4 sessions, this should be the same for both conditions). The regression model will regress treatment condition, gender, location, and baseline PHQ-9 scores on the indicator of whether a patient completed therapy.
2. Randomization checks: Random assignment rules out differences between groups. In other words, differences between groups are due to chance alone and hence should not affect the comparisons between the groups. To assure that randomization worked as intended a series of univariate analyses will compare the treatment groups and control groups in terms of baseline PHQ-9 scores, gender composition and distribution by intervention locations.
3. Comparison of treatment conditions: Assuming that the differences between treatment groups and control are random and neither group were similar in terms of dropout rates, a One-Way Analysis of Variance will be used to compare endline scores for PHQ-9, quality of life and functionality level between the two-intervention groups and post group. Least Significant Difference (LSD) post hoc test will be used

to conduct pair wise comparisons between groups. If the randomization checks describe above reveal a significant difference in PHQ-9 scores, Analysis of Covariance (ANCOVA) will be conducted to adjust the comparisons of end-line PHQ-9 scores by the baseline PHQ-9 scores.

To assess potential influences of gender and location, gender and location on the outcomes, these factors will be included in 2 (gender: male vs. female) 2 (location: Buikwe and Kayunga districts) by 3 (Treatment group: 6 vs. 8 sessions). Any significant interactions of gender or district with treatment group will be probed by comparing the simple main effect of treatment group at each level of gender or district.

4. Analysis of clinically significant effects. Kruskal Wallis nonparametric test will be used to compare the frequency of patients who significant improvement in PHQ-9 scores in treatment groups. As noted above, patients who achieved a change in PHQ-9 scores of five points from baseline to end-line will be considered to show clinically significant improvement. The comparison will be made between the two-treatment group and the control group.

5. **For the qualitative data**, transcribed data will be reviewed by both the project coordinator and a member of the data management committee (DMC) for accuracy and completeness. Thematic analysis will be followed to the analyze data. All the audio recordings will be copied and deleted from the gadgets and uploaded on an online protected cloud for sharing and future use.

Control for potential contamination

Randomization will happen at the individual level (not the community level). Therefore, there will be no need to control for contamination. That said, we shall consider a maximum enrolment of one participant per household. If this is not feasible, we could permit multiple enrolments per household but everyone in the household is automatically assigned to the same arm – i.e., randomization at the household rather than the individual level. This is a little more complicated statistically, but it may be impractical to avoid multiple enrolments per household and will only be explored as the last resort (***See page* 14**).

The stopping for the study rules

Because we expect to fully enroll the study prior to our first participants exiting the study, we have not planned for any interim analysis to assess the need to stop early due to sufficient statistical signal to distinguish the arms, or due to futility. However, the study may stop early for the following reasons:

These will be the major considerations:

1. Safety. If adverse events (AEs) or severe adverse events (SAEs) occur which are potentially related to our study intervention, we will seek IRB and DSMB advice about the need to pause or stop the study. We shall follow a statistical rule of for example any result with $p<0.05$ that the reduction in depression score is higher in TAU could also have the DSMB review AEs that may be associated with the intervention and determine if the study needs to be stopped or paused.
2. Futility. If the study is unable to recruit the target number of participants within the resources available to the study team, or a significant number of participants are dropping out of the study without a clear reason rendering the study unsustainable, the study may need to close due to the financial strain on the study budget making it close to impossible to achieve set targets.
3. COVID-19. If there's a breakout of an infectious disease with clear government mandates dictating lock-down or any other such restrictions that make it a misconduct to continue with in-person treatment.

Bias

To avoid bias, an independent study team will be hired to perform the data collection. Findings will be reviewed by a Research Technical Working Group (RTWG) that will not participate in the data collection and analysis. Questionnaires will be translated to Luganda because it is a language locally spoken in both areas. Some will be left in English depending on the respondent's preference during the study. The questionnaires will be administered by trained, and experienced interviewers using electronic questionnaires loaded on to tablets. However, due to the unreliability of electricity in rural areas some printed versions will be made readily available.

Safety Considerations

Adverse event reporting and harms

Adverse events will be reported to site-specific ethical review boards, the study lead investigators, and other institutional review boards overseeing the study. A clear protocol on how to deal with adverse events will be developed and put in place for everyone involved in the study to follow (see adverse event protocol attached in appendix section)

Confidentiality

Personal information will be collected on consent forms and in a separate demographic sheet that will not be stored with the electronic copies of the data for analyses (see consent forms attached in appendix).

Plans to give access to the full protocol, participant level-data and statistical code

All quantitative data will be made available upon request in de-identified format after publication of the primary study outcomes. Qualitative code summaries will be made available upon request. Because of risk of breach confidentiality, full qualitative recordings or transcripts will not be made publicly available. Sharing of code summaries as an alternative full qualitative transcript is in keeping with qualitative transparency while assuring protection of human subjects in research

Monitoring the Trial

The progress of the trial will be monitored by four committees including the Independent Data monitoring committee (IDMC), Trial management team (TMT), trial steering committee (TSC) and the trial management group (TMG). Details of each are included in the appendix

The Research Coordinator will be responsible for the daily supervision and coordination of the data collection and all research activities. He is responsible for the data to be uploaded from the tablets or computers to the server at the end of each data collection day. He will communicate challenges in data collection and logistics.

The Data Monitoring Committee (DMC) will be responsible for the overall coordination of the research and programme implementation activities. The team will be responsible for data checking, data management and cleaning process, as well as for noticing inconsistencies and challenges along the way. This team will meet regularly to ensure any issues that arise and get to them through the research coordinator are dealt with in a timely manner.

Dissemination of Results

Results will be presented to staff at all the StrongMinds sites in Uganda, USA, and Zambia offices via workshops and disseminated to local communities via community meetings that participated in the study. Results will be disseminated through the global MHPSS community via research reports shared widely and presentations at key conferences, meetings, and sector working groups. Further, results will be disseminated through the international development community via research reports shared widely and presentations at key conferences and meetings. Lastly, results will be disseminated widely through the academic community through publication of peer-reviewed journal articles (we will aim to publish in

open-access journals where possible), and presentation of results at international conference(s).

Trial status

Status: Trial registration ongoing.

References

American Psychiatric Association. (2013). *Diagnostic and statistical manual of mental disorders* (5th ed.). https://doi-org.ezproxy.frederick.edu/10.1176/appi.books.9780890425596

Begg C, Cho M, Eastwood S, Horton R, Moher D, Olkin I, Pitkin R, Rennie D, Schulz KF, Simel D, Stroup DF. Improving the quality of reporting of randomized controlled trials. The CONSORT statement. JAMA. 1996 Aug 28;276(8):637-9. doi: 10.1001/jama.276.8.637. PMID: 8773637.

Federici et al. (2017). World Health Organization disability assessment schedule 2.0: An international systematic review. *Disability and rehabilitation*, 39(23), 2347-2380.

Habtamu et al. (2017). Validation of the World Health Organization Disability Assessment Schedule in people with severe mental disorders in rural Ethiopia. *Health and quality of life outcomes,* 15(1), 1-11.

Health data (2021)**.** New Global Burden of Disease analyses show depression and anxiety among the top causes of health loss worldwide, and a significant increase due to the COVID-19 pandemic. Retrieved on 12/09/2022 from https://www.healthdata.org/acting-data/new-ihme-analyses-show-depression-and-anxiety-among-top-causes-health-burden-worldwide

Kaggwa MM, Namatanzi B, Kule M, Nkola R, Najjuka SM, al Mamun F, Hosen I, Mamun MA, Ashaba S. Depression in Ugandan Rural Women Involved in a Money Saving Group: The Role of Spouse's Unemployment, Extramarital Relationship, and Substance Use. *Int J Womens Health*.2021;13:869-878 https://doi.org/10.2147/IJWH.S323636

Keyser et al. (2022). Function and disability status among women with fistula using WHODAS2. 0: A descriptive study from Rwanda and Democratic Republic of Congo. International *Journal of Gynecology & Obstetrics,* 157(2), 277-282.

Lewandowski, R. E., Bolton, P. A., Feighery, A., Bass, J., Hamba, C., Haroz, E., … Verdeli, H. (2016). Local perceptions of the impact of group interpersonal psychotherapy in rural Uganda. *Global Mental Health*, *3*(4), e23. https://doi.org/10.1017/gmh.2016.15

Lubinga, et al. (2013). Health-related quality of life and social support among women treated for abortion complications in western Uganda. *Health and quality of life*

outcomes, 11(1), 1-8.

Nabunya, P., Damulira, C., Byansi, W. *et al.* Prevalence and correlates of depressive symptoms among high school adolescent girls in southern Uganda. *BMC Public Health* **20**, 1792 (2020). https://doi.org/10.1186/s12889-020-09937-2

Nolens et al. (2018). Birthing experience and quality of life after vacuum delivery and second-stage caesarean section: a prospective cohort study in Uganda. *Tropical Medicine & International Health*, 23(8), 914-922.

Ovuga, E. (2005). The prevalence of depression in two districts of Uganda, 439–445. https://doi.org/10.1007/s00127-005-0915-0

Scholten et al. (2011). Health and functional status among older people with HIV/AIDS in Uganda*. BMC Public Health,* 11(1), 1-10.

UBOS. (2020). 2020 Statistical Abstract.

Vancampfort et al. (2021). The efficacy of a lay health workers–led physical activity counselling program in patients with HIV and mental health problems: a real-world intervention from Uganda. AIDS care, 33(9), 1189-1195.

World Health Organization. (2011). Chapter 6: Sexual Violence. *World Report on Violence and Health*, *26*(5), 608–630. https://doi.org/10.1891/0886-6708.26.5.608

Appendices:

- Schedule of Activities
- Budget
- Questionnaires/instruments
- Recruitment materials
- **SAE/AE protocol**
- **DSMC meetings**
