# Supplementary material for: Six-Week Problem Area–Concordant vs 8-Week Problem Area–Discordant Group Interpersonal Psychotherapy: A Randomized Clinical Trial
Source: JAMA Netw Open. 2025 Apr 16;8(4):e255242. doi: 10.1001/jamanetworkopen.2025.5242 (PMC12004200; doi:10.1001/jamanetworkopen.2025.5242)
Supplement: Supplement 2. — eMethods. Sample Size, Setting and Recruitment, Treatment, Outcomes, and Data Management eTable 1. Baseline Characteristics of Study Participants Retained to End of Therapy and 3-Month Posttherapy Follow-Up eTable 2. Secondary Outcomes of the Effect of Shortened, Trigger-Concordant Interpersonal Group Psychotherapy on Binary Treatment Response Measures (PHQ-9) eTable 3. Pooled Results From Multiple Imputation Analysis of the Effect of Shortened, Problem Area–Concordant Interpersonal Group Psychotherapy on Depression Scores (PHQ-9) at End of Therapy and 3 Months Post Therapy eTable 4. Pooled Results From Multiple Imputation Analysis of the Effect of Shortened, Problem Area–Concordant Interpersonal Group Psychotherapy on Self-Reported Disability, and Self-Reported Quality of Life in the Domains of Physiological Health, Psychological Health, Social Relationships, and Environment at End of Therapy and at 3 Months Post Therapy eTable 5. Pooled Results From Multiple Imputation Analysis of the Effect of Shortened, Problem Area–Concordant Interpersonal Group Psychotherapy on Binary Treatment Response Measures (PHQ-9) eTable 6. Baseline Characteristics of Study Participants Stratified by Retention Status [file jamanetwopen-e255242-s002.pdf]

Supplementary Online Content

Kasujja R, Birungi P, Bhamidipati K, et al. Six-week problem area–concordant vs 8-week problem area–discordant group interpersonal psychotherapy: a randomized clinical trial. *JAMA Netw Open.* 2025;8(4):e255242. doi:10.1001/jamanetworkopen.2025.5242

eMethods. Sample Size, Setting and Recruitment, Treatment, Outcomes, and Data Management

eTable 1. Baseline Characteristics of Study Participants Retained to End of Therapy and 3-Month Posttherapy Follow-Up

eTable 2. Secondary Outcomes of the Effect of Shortened, Trigger-Concordant Interpersonal Group Psychotherapy on Binary Treatment Response Measures (PHQ-9)

eTable 3. Pooled Results from Multiple Imputation Analysis of the Effect of Shortened, Problem Area–Concordant Interpersonal Group Psychotherapy on Depression Scores (PHQ-9) at End of Therapy and 3 Months Post Therapy

eTable 4. Pooled Results from Multiple Imputation Analysis of the Effect of Shortened, Problem Area–Concordant Interpersonal Group Psychotherapy on Self-Reported Disability, and Self-Reported Quality of Life in the Domains of Physiological Health, Psychological Health, Social Relationships, and Environment at End of Therapy and at 3 Months Post Therapy

eTable 5. Pooled Results from Multiple Imputation Analysis of the Effect of Shortened, Problem Area–Concordant Interpersonal Group Psychotherapy on Binary Treatment Response Measures (PHQ-9)

eTable 6. Baseline Characteristics of Study Participants Stratified by Retention Status

This supplementary material has been provided by the authors to give readers additional information about their work.

© 2025 Kasujja R et al. *JAMA Network Open*.

eMethods. Sample Size, Setting and Recruitment, Treatment, Outcomes, and Data Management

Part A: Sample size

This study was powered to test the hypothesis that six-week problem-area-concordant IPT-G is non-inferior to eight-week problem-area-discordant IPT-G (standard-of-care) assuming a non-inferiority margin of one point on the PHQ-9, $\alpha = 0.05$, and $\beta = 0.20$ (i.e., 80% power).

Assumptions regarding outcome scores for power analysis were approximated using routine outcomes data from 8,800 eight-week problem-area-discordant IPT-G clients receiving IPT-G in Uganda. From these data, the following assumptions were used for power calculation: standard deviation of 5.0 PHQ-9 points in post-treatment PHQ-9 scores; within-subject correlation of 0.02; and attribution of 20%. The final estimated sample size requirement was approximately 150 participants per study arm (300 participants total).

Part B: Setting and Recruitment

Recruitment occurred in Buikwe and Kayunga, two districts in central Uganda. Both districts are rural with high poverty rates. The primary local language spoken in both districts is Luganda. The main economic activity in the districts is smallholder farm work. Though located in the same region of Uganda, the two districts are geographically distant enough that travel from one district to the other is not common.

Ugandan districts are sub-divided into counties, and counties are further sub-divided into sub-counties. Buikwe has eleven sub-counties; Kayunga has nine. Two sub-counties from Buikwe and two sub-counties from Kayunga were selected for study recruitment. Participants received therapy within their sub-county to minimize travel-related burden of study participation.

Four mental health facilitators (MHFs), two in each district, were trained to deliver therapy (both six-week and 8-week) to the participants. These facilitators were often locals with an interest in the role, who may have participated in IPT-G themselves. These MHFs were randomly assigned to each district. MHFs delivering the intervention received specific training in the form of an interactive workshop - including demonstrations and role-play – from the investigators. During this training, the focus was on building confidence among MHFs and equipping them with knowledge to face challenges such as time constraints. Practice sessions were carried out by MHFs before delivery of therapy to the study participants, to boost confidence and fidelity to the treatment. Throughout the study, there was ongoing contact between the MHFs and the researchers to monitor any difficulties and during the implementation of the intervention.

For generalizability, study recruitment followed standard procedures used to deliver IPT-G at scale by StrongMinds International. Village health teams performed community sensitization, mobilization, and brief pre-screening of interested residents to assess whether any depression symptoms were present (>0 on any of the first four questions of the PHQ-9). Study staff then conducted individual screening (referred to in StrongMinds routine practice as a pre-group interview) to assess eligibility, including administering the PHQ-9 and assessing depression problem areas through discussion of their individual circumstances.

Part C: Adapted Six-Week Problem-Area-Concordant IPT-G

Initial Phase – 1 session

The session focuses on psychoeducation about depression and setting expectations about what future sessions will cover. The facilitator helps to establish ground rules such as mutual respect and confidentiality, and to clarify treatment goals. The primary objectives of this session are informing participants about depression symptoms, set expectations for therapy, and lay the foundation for social support to be provided.

Working phase – 4 sessions in problem-area-concordant groups, 6 sessions in problem-area-discordant groups

In these sessions, each participant sets goals and employs IPT strategies to reduce their depression symptoms. Strategies are specific to the depression problem area, but techniques typically include decision analysis, communication analysis, linking weekly events to symptoms, and role-playing during therapy. Facilitators administer homework to each participant based on their chosen goals to reduce their symptoms. Each week, participants update the group leader about their depression symptoms using verbal descriptions and visual aids. Participants practice linking mood to events and events to mood and iteratively refine their goals to find sustainable solutions to their underlying problems.

Termination – 1 session

This session focuses on reflecting on the group experience, reviewing goals and progress for each participant, assessing changes in depression symptoms, and planning for how participants will respond to depression symptoms in the future. Participants review the strategies learned over the IPT-G sessions and discuss how they can continue to apply the strategies in their day-to-day lives. Participants are given information on where to seek support in case of a relapse.

© 2025 Kasujja R et al. *JAMA Network Open*.

Part D: Details of Outcome Measures

The Patient Health Questionnaire 9 (PHQ-9) is a nine-item instrument covering the DSM-IV depressive disorder criteria that is routinely used IPT-G treatment eligibility and monitoring in Uganda. PHQ-9 scores range from 0 to 27, with each symptom scored as follows: 0=Not all, 1=Several days, 2=More than half the days, and 3=Nearly every day. A score of ≥10 corresponds well to probable depression with high sensitivity, specificity, and reproducibility across settings.

The WHO Disability Assessment Schedule 2.0 (WHODAS 2.0) is a 12-item questionnaire regarding the difficulty of carrying out activities in the six domains of cognition, mobility, self-care, getting along with people, life activities (household and work), and participation. It was developed by WHO for assessing health and disability in general populations and in specific groups with a range of different mental and physical conditions, including in the context of clinical trials. Scores range from 12 to 60, with each item scored as follows: 1=None, 2=Mild, 3=Moderate, 4=Severe, and 5=Extreme or cannot do. Scores were totaled within each domain as per the WHODAS 2.0 manual.

The WHO Quality of Life –BREF (WHOQOL-BREF) is a 26-item questionnaire regarding quality of life in the four domains of physical, psychological, social and environment, plus two questions regarding the individual's perception of their overall quality of life and health. The instrument was developed by the WHO as an abbreviated version of the WHOQOL-100. Items were scored on a 5-point Likert scale. Negative items were reverse coded. Mean scores from each domain were multiplied by 4 to make them comparable to domain scores from the WHOQOL-100, then transformed to the 0–100 scale, as per the WHOQOL-BREF manual.

Part E: Data management

Hard copies of data collected during the study were stored in locked cabinets at the StrongMinds Global Office in Kampala, Uganda. Electronic data were entered by research assistants using tablets programmed with must-enter variables, range checks, repeat variables, and conditional jumps. Data quality was checked daily by a study coordinator. De-identified data were stored on a secured research data storage system accessible by the trial management committee.

© 2025 Kasujja R et al. *JAMA Network Open*.

eTable 1. Baseline Characteristics of Study Participants Retained to End of Therapy and 3-Month Posttherapy Follow-Up

Balance of baseline characteristics was maintained after attrition.

Sociodemographic Characteristics	End of Therapy			3 Months Post Therapy		
	**8-week arm** n(%)	**6-week arm** n(%)	**p-value**	**8-week arm** n(%)	**6-week arm** n(%)	**p-value**
**Total (N_1= 321, N_2 = 292)**	165 (51.4%)	156 (48.6%)		147 (50.3%)	145 (49.7%)	
**District**			0.5			0.4
Buikwe	80 (48.5%)	82 (52.6%)		65 (44.2%)	72 (49.7%)	
Kayunga	85 (51.2%)	74 (47.4%)		82 (55.8%)	73 (50.3%)	
**Baseline Score (mean ± SD)**	16.75 ± 4.30	17.60 ± 4.36	0.086	16.70 ± 4.36	17.70 ± 4.38	0.052
**Baseline Severity**			0.3			0.2
Mild	0	0		0	0	
Moderate	64 (38.8%)	49 (31.4%)		60 (40.8%)	45 (31.0%)	
Moderately Severe	57 (34.5%)	54 (34.6%)		48 (32.7%)	49 (33.8%)	
Severe	44 (26.7%)	53 (34.0%)		39 (26.5%)	51 (35.2%)	
**Baseline WHODAS 2.0 (mean ± SD)**	20.99 ± 8.35	21.08 ± 7.84	0.8	20.98 ± 8.15	21.39 ± 7.94	0.6
**Baseline WHOQOL-BREF (mean ± SD)**	46.84 ± 8.58	47.44 ± 8.80	0.4	47.03 ± 8.52	47.20 ± 8.89	0.8
**Baseline WHOQOL-BREF Domains (mean ± SD)**						
Physiological	51.02 ± 18.49	51.69 ± 19.05	0.5	50.58 ± 18.68	50.57 ± 18.93	0.8
Psychological	53.11 ± 15.38	55.93 ± 15.69	0.11	53.51 ± 14.70	55.72 ± 15.72	0.2
Social	40.96 ± 22.02	41.61 ± 23.22	0.6	41.38 ± 22.27	41.90 ± 23.47	0.7
Environmental	47.67 ± 16.69	47.26 ± 17.29	>0.9	48.43 ± 16.57	46.81 ± 17.44	0.5
**Age (mean ± SD)**	42.52 ± 16.90	42.21 ± 13.29	0.7	43.76 ± 17.02	42.94 ± 13.24	>0.9
**Gender**			0.4			0.5
Male	9 (5.5%)	12 (7.7%)		8 (5.4%)	11 (7.6%)	
Female	156 (94.5%)	144 (92.3%)		139 (94.6%)	134 (92.4%)	
**Marital Status**			0.4			0.3
Single	3 (1.8%)	7 (4.5%)		3 (2.0%)	7 (4.8%)	
Cohabiting	58 (35.2%)	48 (30.8%)		49 (33.3%)	42 (29.0%)	
Married	46 (27.9%)	41 (26.3%)		46 (31.3%)	41 (28.3%)	
Separated	21 (12.7%)	29 (18.6)		15 (10.2%)	24 (16.6%)	
Widowed	37 (22.4%)	31 (19.9%)		34 (23.1%)	31 (21.4%)	
**Education Level**			0.9			>0.9
Did not attend school	35 (21.2%)	36 (23.1%)		32 (21.8%)	36 (24.8%)	
Lower primary	45 (27.3%)	37 (23.7%)		41 (27.9%)	37 (25.5%)	
Upper primary	60 (36.4%)	61 (39.1%)		51 (34.7%)	51 (35.2%)	
Secondary or higher	25 (15.2%)	22 (14.1%)		23 (15.6%)	21 (14.5%)	
**Number of Children**			0.12			0.14
None	9 (5.5%)	4 (2.6%)		8 (5.4%)	4 (2.8%)	
One child	15 (9.1%)	6 (3.9%)		11 (7.5%)	4 (2.8%)	
2-4 children	36 (21.8%)	41 (26.3%)		30 (20.4%)	38 (26.2%)	
5+ children	105 (63.6%)	105 (67.3%)		98 (66.7%)	99 (68.3%)	
**Trigger Groups**						
Disagreement	59 (35.8%)	55 (35.3%)	>0.9	55 (37.4%)	52 (35.9%)	0.8
Grief	27 (16.4%)	29 (18.6%)	0.6	24 (16.3%)	29 (20.0%)	0.4
Isolation	10 (6.1%)	6 (3.9%)	0.4	9 (6.1%)	5 (3.4%)	0.3
Life change	137 (83.0%)	140 (89.7%)	0.08	122 (83.3%)	130 (89.7%)	0.10
**Number of Problem Areas**			0.15			0.2
One problem area only	99 (60.0%)	81 (51.9%)		86 (58.5%)	73 (50.3%)	
Two or more problem areas	66 (40.0%)	75 (48.1%)		61 (41.5%)	72 (49.7%)	

© 2025 Kasujja R et al. *JAMA Network Open*.

eTable 2. Secondary Outcomes of the Effect of Shortened, Trigger-Concordant Interpersonal Group Psychotherapy on Binary Treatment Response Measures (PHQ-9)

Treatment response measures are binary outcomes of ≥5-point drop in Patient Health Questionnaire 9 (PHQ-9) score indicating clinically significant symptom reduction, ≥10-point drop in PHQ-9 score indicating large symptom reduction, and ≥50% reduction in PHQ-9 score.

**PHQ-9 binary treatment response measures**	**End-of-therapy**		**Three months post-therapy**	
	**Odds ratio (95% CI)**	**p-value**	**Odds ratio (95% CI)**	**p-value**
≥5 Point Score Improvement	1.27 (0.28, 5.75)	0.759	3.19 (0.84, 12.12)	0.089
≥10 Point Score Improvement	2.79 (1.40, 5.56)	0.003	1.90 (0.85, 4.22)	0.063
≥50% Score Improvement	2.16 (0.73, 6.36)	0.163	2.37 (0.81, 6.99)	0.117

© 2025 Kasujja R et al. *JAMA Network Open*.

eTable 3. Pooled Results From Multiple Imputation Analysis of the Effect of Shortened, Problem Area–Concordant Interpersonal Group Psychotherapy on Depression Scores (PHQ-9) at End of Therapy and 3 Months Post Therapy

Effect of randomization to the six-week problem-area-concordant arm are reported as fixed effects (Time*Arm) in a linear mixed effects model with clustering at the therapy group level and individual-level random intercepts. Results (beta coefficients) are reported in units of score change on the PHQ-9.

**PHQ-9 score linear mixed effects model**	**End-of-therapy**		**Three months post-therapy**	
	**β estimate (95% CI)**	**p-value**	**β estimate (95% CI)**	**p-value**
Time × Arm (6-wk vs. 8-wk arm after treatment)	-1.82 (-3.00, -0.65)	0.002	-1.74 (-2.94, -0.54)	0.005

© 2025 Kasujja R et al. *JAMA Network Open*.

eTable 4. Pooled Results From Multiple Imputation Analysis of the Effect of Shortened, Problem Area–Concordant Interpersonal Group Psychotherapy on Self-Reported Disability, and Self-Reported Quality of Life in the Domains of Physiological Health, Psychological Health, Social Relationships, and Environment at End of Therapy and at 3 Months Post Therapy

Disability and quality-of-life results are reported as fixed effects in a linear mixed effects model, with beta coefficients in units of score change from baseline on the WHO Disability Assessment Schedule 2.0 (WHODAS) and WHO Quality of Life– BREF (WHOQOL) questionnaires.

**Disability and quality-of-life score changes**	**End-of-therapy**		**Three months post-therapy**	
	**β estimate (95% CI)**	**p-value[1]**	**β estimate (95% CI)**	**p-value[1]**
**WHODAS Scores**				
Time × Arm (6-wk vs. 8-wk arm after treatment)	-2.44 (-4.15, -0.73)	0.025	-0.20 (-1.95, 1.54)	1.000
**WHOQOL - Physiological**				
Time × Arm (6-wk vs. 8-wk arm after treatment)	5.21 (0.26, 10.17)	0.195	-0.13 (-5.57, 5.32)	1.000
**WHOQOL - Psychological**				
Time × Arm (6-wk vs. 8-wk arm after treatment)	0.87 (-3.10, 4.84)	1.000	-0.70 (-4.73, 3.34)	1.000
**WHOQOL - Social**				
Time × Arm (6-wk vs. 8-wk arm after treatment)	1.73 (-3.46, 6.92)	1.000	-1.26 (-6.46, 3.94)	1.000
**WHOQOL - Environmental**				
Time × Arm (6-wk vs. 8-wk arm after treatment)	4.49 (0.33, 8.65)	0.175	1.98 (-2.40, 6.35)	1.000

[1]*Bonferroni-adjusted p-value*

© 2025 Kasujja R et al. *JAMA Network Open*.

eTable 5. Pooled Results From Multiple Imputation Analysis of the Effect of Shortened, Problem Area–Concordant Interpersonal Group Psychotherapy on Binary Treatment Response Measures (PHQ-9)

Treatment response measures are binary outcomes of ≥5-point drop in Patient Health Questionnaire 9 (PHQ-9) score indicating clinically significant symptom reduction, ≥10-point drop in PHQ-9 score indicating large symptom reduction, and ≥50% reduction in PHQ-9 score.

PHQ-9 binary treatment response measures	End-of-therapy		Three months post-therapy	
	Odds ratio (95% CI)	p-value[1]	Odds ratio (95% CI)	p-value[1]
≥5 Point Score Improvement	0.31 (-1.20, 4.62)	0.687	1.06 (-0.13, 2.24)	0.081
≥10 Point Score Improvement	1.04 (0.35, 1.73)	0.004	0.58 (-0.22, 1.38)	0.153
≥50% Score Improvement	0.80 (-0.28, 1.89)	0.148	0.77 (-0.21, 1.75)	0.126

© 2025 Kasujja R et al. *JAMA Network Open*.

eTable 6. Baseline Characteristics of Study Participants Stratified by Retention Status

Baseline Characteristics	Retained in Analysis	Lost to Follow-up	p-value
**Total (N = 328)**	292 (89.02%)	36 (10.98%)	
**District**			0.001
Buikwe	137 (47%)	27 (75%)	
Kayunga	155 (53%)	9 (25%)	
**Baseline PHQ-9 Score (mean ± SD)**	17.20 ± 4.39	17.06 ± 4.08	>0.9
**Baseline Depression Severity**			0.13
Mild	0	0	
Moderate	105 (36%)	9 (25%)	
Moderately Severe	97 (33%)	18 (50%)	
Severe	90 (31%)	9 (25%)	
**Baseline WHODAS 2.0 (mean ± SD)**	21.18 ± 8.04	20.75 ± 9.29	0.4
**Baseline WHOQOL-BREF (mean ± SD)**	47.11 ± 8.69	46.54 ± 8.91	0.5
**Baseline WHOQOL-BREF Domains (mean ± SD)**			
Physiological	50.57 ± 18.77	57.54 ± 16.51	0.046
Psychological	54.61 ± 15.23	52.66 ± 18.45	0.3
Social	41.64 ± 22.83	35.88 ± 20.00	0.13
Environmental	47.62 ± 17.00	44.79 ± 16.95	0.3
**Age (mean ± SD)**	43.35 ± 15.24	33.33 ± 11.35	<0.001
**Gender**			0.043
Male	19 (6.5%)	30 (83%)	
Female	273 (93%)	6 (17%)	
**Marital Status**			0.007
Single	10 (3.4%)	1 (2.8)	
Cohabiting	91 (31%)	16 (44%)	
Married	87 (30%)	5 (14%)	
Separated	39 (13%)	11 (31%)	
Widowed	65 (22%)	3 (8.3%)	
**Education Level**			0.006
Did not attend school	68 (23%)	3 (8.3%)	
Lower primary	78 (27%)	5 (14%)	
Upper primary	102 (35%)	23 (64%)	
Secondary or higher	44 (15%)	5 (14%)	
**Number of Children**			0.016
None	12 (4.1%)	1 (2.8%)	
One child	15 (5.1%)	7 (19%)	
2-4 children	68 (23%)	10 (28%)	
5+ children	197 (67%)	18 (50%)	
**Problem Areas**			
Disagreement	107 (37%)	10 (28%)	0.3
Grief	53 (18%)	3 (8.3%)	0.14
Isolation	14 (4.8%)	4 (11%)	0.12
Life change	252 (86%)	31 (86%)	>0.9
**Number of Problem Areas**			0.2
One problem area only	159 (54%)	24 (67%)	
Two or more problem areas	133 (46%)	12 (33%)	

© 2025 Kasujja R et al. *JAMA Network Open*.
