# Supplementary material for: Six-Week Problem Area–Concordant vs 8-Week Problem Area–Discordant Group Interpersonal Psychotherapy: A Randomized Clinical Trial
Source: JAMA Netw Open. 2025 Apr 16;8(4):e255242. doi: 10.1001/jamanetworkopen.2025.5242 (PMC12004200; doi:10.1001/jamanetworkopen.2025.5242)
Supplement: Supplement 4. — Data Sharing Statement [file jamanetwopen-e255242-s004.pdf]

Data Sharing Statement

Kasujja. Six-Week Problem Area–Concordant vs 8-Week Problem Area–Discordant Group Interpersonal Psychotherapy. *JAMA Netw Open*. Published April 16, 2025. doi:10.1001/jamanetworkopen.2025.5242

Data
Additional Information: PACTR202306771120632
Data available: Yes
Data types: Deidentified participant data
How to access data: A de-identified dataset will be published as Supplementary Material to the manuscript. It is provided in the submitted materials for peer review.
When available: With publication

Supporting Documents
Document types: Other (please specify)
Additional Information: Trial protocol
How to access documents: Our approved clinical trial protocol is provided as Supplementary Material and will be published with the paper, if accepted.
When available: With publication

Additional Information
Who can access the data: Data will be available to anyone who wishes to access it.
Types of analyses: The data can be used to reproduce results reported in the manuscript.
Mechanisms of data availability: The data will be made available online as Supplementary Materials. Users may reach out to the corresponding author for support if needed. Use does not require a signed data access agreement.
